# Global skin disease burden: A comparative study of dermatologic disease in Enugu State, Nigeria

**DOI:** 10.1016/j.jdin.2025.02.006

**Published:** 2025-04-17

**Authors:** William S. Smith, Summer V. Morrissette, Robert H. Burrow, Maureen N. Offiah, Robert T. Brodell, Thomas O. Nnaji, Vinayak K. Nahar

**Affiliations:** a Department of Dermatology, School of Medicine, University of Mississippi Medical Center, Jackson, Mississippi; b Department of Internal Medicine Ebonyi State University Abakaliki, Nigeria; c Department of Preventive Medicine, School of Medicine/John D. Bower School of Population Health, University of Mississippi Medical Center, Jackson, Mississippi

In 2017, the University of Mississippi Medical Center Department of Dermatology collaborated with Enugu State University Teaching Hospital to establish a medical mission in Southeastern Nigeria. This study assesses the prevalence of dermatologic diseases in Nigeria, reviews previously published data, discusses the epidemiological significance of these findings, and emphasizes the importance of training local medical students during medical mission trips to provide sustainable benefits.

Total of 467 diagnoses were recorded from 1005 patients seen at the teaching hospital and in 3 rural clinics by 4 faculty members who spent successive weeks with our chief resident, who saw clinic patients the entire month. Diagnostic categories were delineated and compared with reported values from previous Nigerian surveys conducted from 1994 to 2007. Bedside teaching and 10 lectures delivered to the entire medical school student body ensured this work had sustainable benefits.

Total of 1005 patients with 467 diagnoses were seen at the teaching hospital and in 3 rural clinics. Infections/infestations and eczematous dermatitis are the most common conditions in our experience, and previous studies with follicular disorders, skin tumors, and pruritus/hives are also well represented. (Table I).^1^, ^2^, ^3^, ^4^, ^5^

**Table I:** Prevalence of skin disease groups across Nigeria

Skin diseases	2017	2006-07^1^	2000-05^2^	1999-01^3^	1999-01^4^	1994-98^5^
Infections or infestations	34.5%	20.1%	21.7%	19.1%	44.4%	21.4%
Eczematous dermatitis	23.3%	20.9%	35.2%	20.3%	14.1%	30.2%
Follicular disorders	10.9%	7.7%	8.1%	13.7%	7.0%	5.7%
Skin tumors	9.4%	7.4%	2.7%	5.0%	4.4%	-
Urticaria or pruritus	6.0%	6.6%	7.9%	9.4%	6.0%	9.0%
Papulosquamous disorders	4.9%	9.0%	4.9%	10.7%	6.4%	5.9%
Pigmentary disorders	4.5%	5.0%	3.9%	10.4%	6.0%	5.4%
Connective tissue disorders	1.1%	1.7%	0.9%	1.5%	2.0%	2.6%
Hair disorders	1.1%	1.5%	2.3%	1.3%	5.6%	-
Drug eruptions	0.4%	4.0%	2.7%	1.1%	1.2%	2.3%
Sexually transmitted diseases	0.2%	7.9%	4.3%	5.4%	-	-
Other	3.6%	8.1%	5.5%	2.1%	2.8%	17.7%

This study illustrates the prevalence of skin disease in Nigeria’s Enugu State and identifies similar patterns in previous studies over decades. The highest prevalence category observed in each study was infectious/infestations. This may be related to overcrowding, poor living and sanitary conditions, and malnutrition.^1^^,^^2^^,^^4^ In addition, the high humidity and warm tropical climate have been shown to contribute to outbreaks of infestations and infections in Nigeria, Jamaica, and Ethiopia.^4^ The high prevalence of drug-resistant scabies and the inability to access newer, more efficacious treatments may play a role.^4^ Monkeypox was one infectious condition none of us had seen (Fig 1, Supplementary Figures 1-8 available on Mendeley at https://data.mendeley.com/datasets/y8t8r8hv24/1). Finally, rural Nigerian diseases are certainly impacted by limited access to medical care and resources when compared with cities, highlighting the need for investments to impact so many of these treatable conditions.

**Fig 1:**
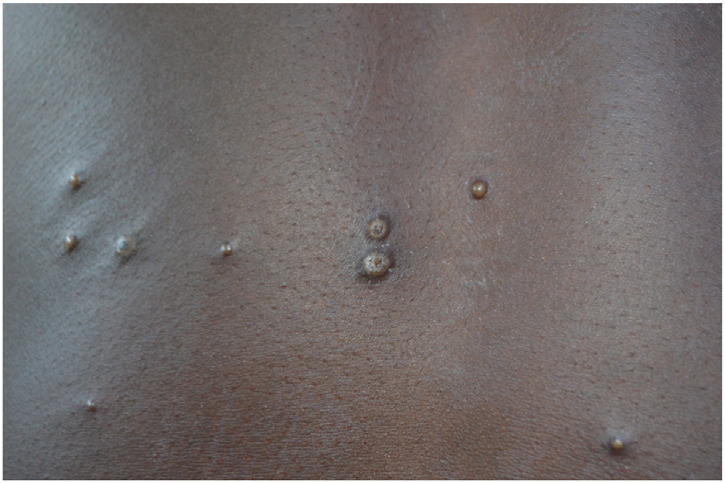
A 22-year-old male with 1-week history of blistering, papular and nodular lesions on the face and back after exposure to animals in the bush. These 2-mm to 10-mm keratotic papulo-vesciular lesions were present on the back. Monkeypox was confirmed by serology.

Eczematous dermatitis was the second most observed skin disease in the clinics we staffed and was in the top 2 most frequent diagnoses seen in the previous Nigerian studies from 1994 to 2007. It has been postulated that industrialization with exposure to chemical irritants, rubbers, dyes, cosmetic products, and more over the counter medications, such as topical antimicrobial and steroidal creams may be responsible for the high prevalence of ecema.^1^ Follicular disorders, like acne, ranked in the top 3 categories in all studies (Table I^1^, ^2^, ^3^, ^4^, ^5^). Skin tumors included nevi, seborrheic keratoses, epidermal nevus, neurofibroma, squamous cell carcinoma, and basal cell carcinoma and were similar to previous descriptions.

Dermatologists have the expertise to care for and treat skin disease around the world. There was a high prevalence of skin diseases in Nigeria, yet Enugu State, with 4.5 million citizens, had 7 dermatologists and 1 resident. Rewards for volunteers include an opportunity to see incredible pathology (Supplementary material available on Mendeley at https://data.mendeley.com/datasets/y8t8r8hv24/1), while patients are treated and medical students are taught. Thus, medical mission trips can focus dermatologic expertise in developing countries while educating physicians to carry on after the trip has ended.

## Conflicts of interest

Dr Brodell is a principal investigator for clinical trials (Novartis, Lilly, and Sanofi), the Corevitas psoriasis biologic registry, and owns stock in Veradermics Inc. Drs Morrissette, Nahar, Burrow, Offiah, Sullivan, Nnaji, and Mr. Smith have no financial interests to disclose.
